# 16S rRNA Gene Diversity in the Salt Crust of Salar de Uyuni, Bolivia, the World’s Largest Salt Flat

**DOI:** 10.1128/MRA.00374-20

**Published:** 2020-05-21

**Authors:** Wolf T. Pecher, Fabiana L. Martínez, Priya DasSarma, Daniel Guzmán, Shiladitya DasSarma

**Affiliations:** a Institute of Marine and Environmental Technology, Department of Microbiology and Immunology, University of Maryland School of Medicine, Baltimore, Maryland, USA; b Yale Gordon College of Arts and Sciences, University of Baltimore, Baltimore, Maryland, USA; c Instituto de Investigaciones para la Industria Química, Consejo Nacional de Investigaciones Científicas y Técnicas, Universidad Nacional de Salta, Salta, Argentina; d Centro de Biotecnología, Faculty of Sciences and Technology, Universidad Mayor de San Simón, Cochabamba, Bolivia; Portland State University

## Abstract

Salar de Uyuni is a vast, high-altitude salt flat in Bolivia with extreme physico-geochemical properties approaching multiple limits of life. Evidence for diverse halophilic bacteria and archaea was found in its surface and near-surface salt crust using 16S amplicon analysis, providing a snapshot of prokaryotic life.

## ANNOUNCEMENT

Salar de Uyuni is the world’s largest salt flat, located in the southwestern Altiplano of Bolivia at an altitude of approximately 3,650 m above sea level (1, 2). The geochemical composition of brines and salt crusts (high in lithium, boron, magnesium, potassium, sodium, and chloride) and other physical conditions (high daily temperature fluctuations, UV radiation, and albedo) create a polyextreme environment close to multiple limits of life (3–5). In addition to an understanding of extremophilic life on Earth, characterization of the microbial communities of Salar de Uyuni will contribute to determining the potential habitability of other planets, as well as new biotechnology applications (6–9). To date, however, few microbial genomic studies have been conducted (10–13). This study provides an initial snapshot of microbial diversity in the crust.

Samples were collected in March 2015 from a remote site (20°33′28.58ʺS, 67°12′29.56ʺW) in Salar de Uyuni from the surface salt crust (SSC) (BOL4) and at a depth of 5 to 15 cm (near-surface salt crust [NSSC] [BOL3]). DNA was extracted with the PowerLyzer PowerSoil DNA extraction kit (MO BIO Laboratories, Inc., Carlsbad, CA). Library construction and 16S amplicon sequencing of the V3 to V4 region were performed on a MiSeq platform according to the manufacturer’s recommendations (Illumina, Inc., San Diego, CA) using the primers Bakt_341F and Bakt_805R (14–16).

Raw reads were processed with mothur (v1.39.5), and sequence data were aggregated with R (v3.4.1) (16–19) (https://www.mothur.org/wiki/MiSeq_SOP). Paired-end sequencing generated 213,999 raw reads (median length, 459 bp [range, 35 to 600 bp]). Reads were assembled with a quality score threshold of 20. Sequences longer than 475 bp and those with ambiguities and homopolymers (>8 bp), as well as chimeras, were removed, and sequences were aligned against the SILVA small subunit (SSU) Ref NR 99 database (v132) (20). Based on analysis using mothur, sequences with at least 97% similarity were binned into operational taxonomic units and classified (using a pseudobootstrap value of 80) against the reference database trimmed to positions 201 to 1000 of the 16S sequence of Escherichia coli (GenBank accession number J01859.1). Singletons were removed and only prokaryotic sequences were retained, resulting in 21,636 (SSC) and 3,625 (NSSC) sequences with a median length of 457 bp (range, 419 to 466 bp).

Archaea constituted 13.47% of the SSC sequences and 11.50% of the NSSC sequences, and bacteria constituted 86.53% and 88.50%, respectively. For archaea, all sequences were classified at the phylum level and 96.57% (SSC) and 91.85% (NSSC) at the genus level; 98.73% (SSC) and 93.29% (NSSC) were *Euryarchaeota*, and 1.27% (SSC) and 6.24% (NSSC) were *Nanoarchaeota*. *Hadesarchaeota* were present only in NSSC (0.48%). The most prevalent genera were *Halonotius* in SSC (53.09%) and *Halodesulfurarchaeum* in NSSC (47.96%) (Table 1). For bacteria, 99.71% (SSC) and 93.95% (NSSC) of sequences were classified at the phylum level and 91.36% (SSC) and 73.72% (NSSC) at the genus level. *Bacteroidetes* (58.25% [SSC] and 11.69% [NSSC]) and *Proteobacteria* (40.63% [SSC] and 73.72% [NSSC]) were most prevalent at the phylum level, and *Salinibacter* in SSC (53.21%) and *Halorhodospira* in NSSC (30.17%) were most prevalent at the genus level (Table 1). These findings represent a snapshot of considerable prokaryotic diversity in the largest salt flat on Earth.

**TABLE 1:** Prevalence of archaeal and bacterial 16S amplicons at the phylum and genus levels in the SSC and NSSC of Salar de Uyuni

Sample and taxonomic category^*a*^	Total no. of sequences	Abundance (%)^*b*^
SSC (BOL 4)		
Archaea		
Phyla		
*Euryarchaeota*	2,877	98.73
*Nanoarchaeota*	37	1.27
*Hadesarchaeota*	0	0.00
Genera		
*Halonotius*	1,547	53.09
*Halorubrum*	542	18.60
*Halobellus*	276	9.47
*Halovenus*	202	6.93
*Natronomonas*	131	4.50
*Halococcus*	22	0.75
A07HB70	19	0.65
*Halodesulfurarchaeum*	18	0.62
*Halapricum*	15	0.51
*Halobacterium*	12	0.41
Bacteria		
Phyla		
*Bacteroidetes*	10,906	58.25
*Proteobacteria*	7,606	40.63
*Patescibacteria*	66	0.35
Genera		
*Salinibacter*	9,962	53.21
*Acinetobacter*	5,181	27.67
*Arhodomonas*	735	3.93
*Brevundimonas*	264	1.41
*Halofilum*	191	1.02
*Delftia*	142	0.76
*Diaphorobacter*	132	0.71
*Salicola*	123	0.66
*Fodinibius*	75	0.40
*Thiohalospira*	38	0.20
NSSC (BOL 3)		
Archaea		
Phyla		
*Euryarchaeota*	389	93.29
*Nanoarchaeota*	26	6.24
*Hadesarchaeota*	2	0.48
Genera		
*Halodesulfurarchaeum*	200	47.96
*Halonotius*	39	9.35
*Halapricum*	34	8.15
*Halobellus*	28	6.71
*Halanaeroarchaeum*	26	6.24
*Halorubrum*	19	4.56
*Halovenus*	8	1.92
*Natronomonas*	8	1.92
*Haloarcula*	5	1.20
“*Candidatus* Haloredivivus”	4	0.96
Bacteria		
Phyla		
*Proteobacteria*	2,365	73.72
*Bacteroidetes*	375	11.69
*Halanaerobiaeota*	194	6.05
Genera		
*Halorhodospira*	968	30.17
*Desulfovermiculus*	351	10.94
*Acinetobacter*	314	9.79
*Arhodomonas*	211	6.58
*Salinibacter*	200	6.23
*Halanaerobium*	194	6.05
*Thiohalorhabdus*	28	0.87
*Thiohalospira*	23	0.72
*Halofilum*	19	0.59
*Limimonas*	15	0.47

a The 3 most prevalent phyla and 10 most prevalent genera for each sample are shown.

b Abundance was calculated based on the total number of sequences in each domain.

### Data availability.

The 16S rRNA gene amplicon data sets are available at NCBI under SRA accession numbers SRX7011107 (NSSC) and SRX7011108 (SSC).
